# Electrochemical Potential Influences Phenazine Production, Electron Transfer and Consequently Electric Current Generation by *Pseudomonas aeruginosa*

**DOI:** 10.3389/fmicb.2017.00892

**Published:** 2017-05-18

**Authors:** Erick M. Bosire, Miriam A. Rosenbaum

**Affiliations:** Institute of Applied Microbiology, Aachen Biology and Biotechnology, RWTH Aachen UniversityAachen, Germany

**Keywords:** *Pseudomonas aeruginosa*, electrode potential, phenazines, bioelectrochemical system, electron transfer, phenazine-1-carboxylic acid

## Abstract

*Pseudomonas aeruginosa* has gained interest as a redox mediator (phenazines) producer in bioelectrochemical systems. Several biotic and abiotic factors influence the production of phenazines in synergy with the central virulence factors production regulation. It is, however, not clear how the electrochemical environment may influence the production and usage of phenazines by *P. aeruginosa*. We here determined the influence of the electrochemical potential on phenazine production and phenazine electron transfer capacity at selected applied potentials from -0.4 to +0.4 V (vs. Ag/AgCl_sat_) using *P. aeruginosa* strain PA14. Our study reveals a profound influence of the electrochemical potential on the amount of phenazine-1-carboxylate production, whereby applied potentials that were more positive than the formal potential of this dominating phenazine (E^° ′^_PCA_ = -0.24 V vs. Ag/AgCl_sat_) stimulated more PCA production (94, 84, 128, and 140 μg mL^-1^ for -0.1, 0.1, 0.2, and 0.3 V, respectively) compared to more reduced potentials (38, 75, and 7 μg mL^-1^ for -0.4, -0.3, and -0.24 V, respectively). Interestingly, *P. aeruginosa* seems to produce an additional redox mediator (with E^° ′^ ∼ 0.052 V) at applied potentials below 0 V, which is most likely adsorbed to the electrode or present on the cells forming the biofilm around electrodes. At fairly negative applied electrode potentials, both PCA and the unknown redox compound mediate cathodic current generation. This study provides important insights applicable in optimizing the BES conditions and cultures for effective production and utilization of *P. aeruginosa* phenazines. It further stimulates investigations into the physiological impacts of the electrochemical environment, which might be decisive in the application of phenazines for electron transfer with *P. aeruginosa* pure- or microbial mixed cultures.

## Introduction

Bioelectrochemical systems (BES) comprise a wide array of technologies that are based on the interaction of microorganisms with electrodes (Schröder et al., 2013). The most common technology is the microbial fuel cell (MFC) in which microbes convert organic materials in, for instance, wastewater into electrical energy. To harness the electrons liberated from oxidation of the organic substrates at an anode, the potentials in BES are set with regard to the terminal electron acceptor at the cathode; mostly oxygen. Microbes are proposed to use different electron transfer strategies to shuttle electrons to the anode: direct electron transfer via *c*-type cytochromes and other redox proteins or cell-like extensions termed nanowires, and mediated electron transfer via endogenous or exogenous soluble redox mediators (Gorby et al., 2006; Schröder, 2007; Marsili et al., 2008; Malvankar et al., 2011; Wrighton et al., 2011). Soluble redox mediators may include phenazines, riboflavins, and quinones produced by *Pseudomonads, Shewanella*, and *Lactococcus*, respectively (Marsili et al., 2008; Pham et al., 2008; Freguia et al., 2009).

Phenazines are promising natural and synthetic redox mediators for enhancing current production in BES, and *Pseudomonas aeruginosa* is one of the most active producers. *P. aeruginosa* has indeed shown potential of being used as the phenazine producer in BES co-cultures, allowing partner organisms to utilize the supplied phenazines for metabolic electron discharge (Rabaey et al., 2005; Pham et al., 2008; Bosire et al., 2016). Phenazine production by *Pseudomonads* is influenced by several biotic and abiotic factors in the ecological niches (van Rij et al., 2004; Mavrodi et al., 2006). Most importantly for BES function, they play important roles in the metabolism of microorganisms in cases where the natural electron acceptor is missing or limiting. In *P. aeruginosa*, which is not able to ferment, pyocyanin (PYO) has been confirmed to contribute in maintaining the cellular redox balance by oxidizing NADH (Pierson and Pierson, 2010). Under anaerobic conditions, PYO redox cycling may enable *P. aeruginosa* to survive (Wang et al., 2010).

There are four well known *P. aeruginosa* phenazines with fairly close redox potentials vs. a Ag/AgCl reference electrode: PCA (-0.24 V), PYO (-0.116 V), 1-hydroxy-phenazine (1-HP; -0.174 V), and phenazine-1-carboxamide (PCN; -0.14 V) [given are formal potentials of standards at the conditions in our bioelectrochemical setups as determined in (Bosire et al., 2016)]. However, they have varying properties and redox reactivities to electron acceptors; suggesting that they may play different roles in, for example, biofilms. PYO reacts more readily with oxygen at neutral pH while PCA and the other phenazines are more reactive to solid electron acceptors like iron oxides and hydroxides (Wang and Newman, 2008). Hence, based on their roles, concentration gradients of the phenazine species might exist in biofilms where oxygen availability-gradients prevail. Considering that the production of phenazines is stimulated by the prevailing environmental factors including oxygen and iron (van Rij et al., 2004), it is probable that *P. aeruginosa* might produce different gradients of these phenazines depending on need or on the electron acceptor potential or properties. So far, all pure or co-culture observations of *P. aeruginosa* in BES research have been performed at one fixed electrode potential to guarantee stable electrochemical conditions (typically +0.2 or +0.3 V vs. RE) (Venkataraman et al., 2010, 2011; Bosire et al., 2016). Therefore, it is an important question whether the applied electrode potential, which determines the redox environment, might influence phenazine production or the phenazine spectrum and their capacity in electron shuttling. For other cases, it was even shown that the applied potential might influence electron transfer strategies of the microorganism (Liu et al., 2010). Thus, for *Shewanella oneidensis*, which is able to employ different electron transfer mechanisms (i.e., direct vs. mediated or a combination), the available potential may influence the use of these mechanisms and subsequently a shift between them. This redox-stimulated switch in electron transfer mechanism is also associated with a change in the level of electric current production (Liu et al., 2010; Lian et al., 2016). Hence, there is great potential in understanding how to correctly poise or regulate the electrodes in BES in order to obtain an optimal metabolic state also for *P. aeruginosa* for most productive phenazine and current production.

Therefore, the aim of this study was to evaluate the cellular physiology, phenazine production, and subsequent electric current generation of *P. aeruginosa* strain PA14 at a broad range of applied electrode potentials ranging from potentials more negative than the phenazine formal potentials (i.e., the electrode could serve as electron donor for phenazine reduction) to common electro-positive redox potentials, which allow for electrochemical oxidation of reduced phenazines. The knowledge gained is expected to be instrumental in optimizing future (co-)cultures for efficient electron transfer in BES applications.

## Materials and Methods

### Strain and Culture Conditions

In this study, *P. aeruginosa* strain PA14 (DSMZ 19882) from the German Collection of Microorganisms and Cell Culture was used. The strain was pre-cultured overnight in AB medium at 37°C and washed three times with equal volume of 0.9% NaCl before being used to inoculate the BES reactors. For BES experiments, strains were cultured in AB medium. Procedure for preparing AB was adopted from Clark and Maaløe (1967). The medium contained the following constituents (per liter): component A: 2.0 g (NH_4_)_2_SO_4_, 6.0 g Na_2_HPO_4_, 3.0 g KH_2_PO_4_, 3.0 g NaC1, 0.011 g Na_2_SO_4_, and component B: 0.2 g MgCl_2_, 0.010 g CaCl_2_ and 0.5 mg FeC1_3_ × 7 H_2_0 (Clark and Maaløe, 1967). The two components were autoclaved separately before mixing them. Glucose was supplied as the carbon and electron donor at a concentration of 20–30 mM (see respective experiments).

### BES Setup and Electrochemical Procedures

A single-chambered bioelectrochemical cell with a three-electrode configuration as described before was used (Bosire et al., 2016). The Ivium-n-Stat potentiostat (Ivium Technologies, Eindhoven, The Netherlands) was used to perform the electrochemical measurements. Chronoamperometric measurement was used to monitor electric current generation at varying potentials. A total of eight potentials ranging from -0.4 V to +0.4 V (vs. Ag/AgCl_sat_.) were tested in individual triplicate or duplicate (see individual experiments) BES setups in batch mode. Replicate reactor runs were started with the same medium batch and inoculum culture at a starting OD of 0.1. Current generation was recoded over the entire growth period at each set potential. Chronoamperometric measurement was interrupted every 23 h to perform a cyclic voltammetry scan similar as published in Bosire et al. (2016). Cyclic voltammetry was performed at a potential range of -0.5 to 0.5 V at a scan rate of 2 mVs^-1^. Coulombic efficiency was calculated as the percentage of the collected charge compared to the charge supplied in the carbon source (glucose: 24 electrons per molecule).

### Biomass Measurement

Cell dry weight was measured at the end of the experiment by carefully scraping off the biofilm around the electrodes and the reactor walls into the culture medium. The whole culture medium was centrifuged at 10,000 rpm for 20 min and the pellet was washed three times with 0.09% NaCl. The pellet was transferred into pre-weighed aluminum dishes and the containers were carefully rinsed to ensure the transfer of all the biomass. The biomass was dried overnight at 120°C and weighed in a moisture analyzer (RADWAG moisture analyzer, Hilden, Germany).

### Analytical Procedures

For phenazine detection and quantification, samples were separated in a waters symmetry column, Symmetry^®^ 5 μm C18 4.6 mm × 250 mm (Waters, Herts, UK) using a Beckman Gold HPLC (Beckman Coulter Inc., Brea, CA, USA) fitted with a photo diode array detector. 0.1% formic acid (pH 5) was used as solvent A and acetonitrile as solvent B at a flow rate of 0.5 mL min^-1^. A linear gradient was run for 28 min as follows: 5 min 10% acetonitrile, 10 min linear gradient to 100% acetonitrile, 10 min 100% acetonitrile, 1 min linear gradient to 10% acetonitrile, and 3 min 10% acetonitrile. Phenazines were separated and detected at their characteristic wavelengths; PYO-319 nm, PCA-366 nm, 1-HP-247 nm, and PCN-366 nm. Stock solutions of PCA and PCN (Princeton Biomolecular) and 1-HP (TCA Europe), were made by dissolving 1000 μg mL^-1^ of the phenazines in dimethyl sulfoxide (DMSO, Sigma-Aldrich). For PYO (Cayman Chemical), the stock solutions were made by dissolving 2500 μg mL^-1^ in 100% ethanol.

For the detection and quantification of carbon sources and metabolites, an organic acid resin column (300 mm × 8 mm polystyrol-divinylbenzol copolymer [PS-DVB], CS-Chromatography) was used to separate the culture supernatants on a Dionex Ultimate 3000 HPLC system (Sunnyvale, CA, USA) equipped with a refractive index (RI-101, Shodex) and UV detector (Ultimate 3000 UV/VIS detector, Dionex). Sulphuric acid (5 mM) was applied as the eluent at a flow rate of 0.8 mL min^-1^ and a temperature of 60°C.

## Results

The most dominating phenazine in all previous work with *P. aeruginosa* PA14 in our group was PCA with a formal potential of -0.24 V (Bosire et al., 2016). We therefore explored applied electrode potentials more negative and more positive than this potential (eight potentials from -0.4 V to +0.4 V). Anodic (oxidative) behavior was expected at potentials above -0.24 V, where PCA can be electrochemically oxidized at the electrode (results grouped as anodic potentials). Below this potential, it was expected that there will be no electron discharge to the electrode using this phenazine (results grouped as cathodic potentials). To understand the influence of the applied potential on the redox mediator production, the phenazines PCA, PYO, PCN, and 1-HP were quantified. Also, the siderophore pyoverdine, which is involved in iron acquisition and might exhibit redox properties, was detected and quantified. While the phenazines PCN and 1-HP were below detection limit in all the cultures, PCA and PYO were detected in varying concentrations in cultures grown at the different potentials.

### Phenazine Production and Current Generation at Cathodic Potentials

**FIGURE 1:**
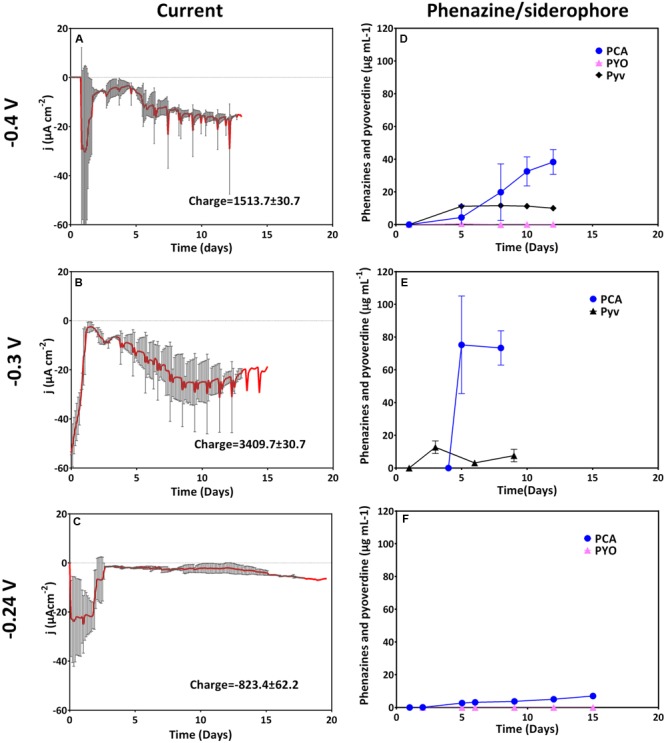
**Current generation and redox mediator production at cathodic (reducing) potentials.** Left row depicts current generation over time and right row depicts redox mediator and siderophore production. From top to bottom is a comparison of current and phenazine/siderophore generation for biological replicates poised at **(A,D)** -0.4 V (*n* = 3), **(B,E)** -0.3 V (*n* = 2), and **(C,F)** -0.24 V (*n* = 3).

At the three most electro-negative applied potentials (-0.4, -0.3, and -0.24 V), an initial cathodic (reducing) current (days 1–2) most likely reflects abiotic oxygen reduction at the cathode. With biological oxygen consumption in the reactor, this initial current subsides around day 2. Thereafter, with culture growth, biotic cathodic currents were observed at these low potentials including the formal potential (-0.24 V) of the dominatingly produced phenazine, PCA (**Figure 1**). At -0.4 V, an accelerating reduction current was generated at the working electrode over 12 days, at -0.3 V this reduction current increased even faster. It therefore appears that the negative current after day 2 generated can be reconciled with growth.

PCA strongly dominated at all applied potentials even though the concentration was very low when the electrode was poised right at the formal potential of PCA (-0.24 V). Concentrations of PYO were below or very close to the detection limit for all three potentials. Varying low concentrations of pyoverdine were produced at the different cathodic potentials (up to 11 μg mL^-1^ for -0.4 V and 15.6 μg mL^-1^ for -0.3 V, respectively (**Figure 1**).

### Phenazine Production and Current Generation at Anodic Potentials

**FIGURE 2:**
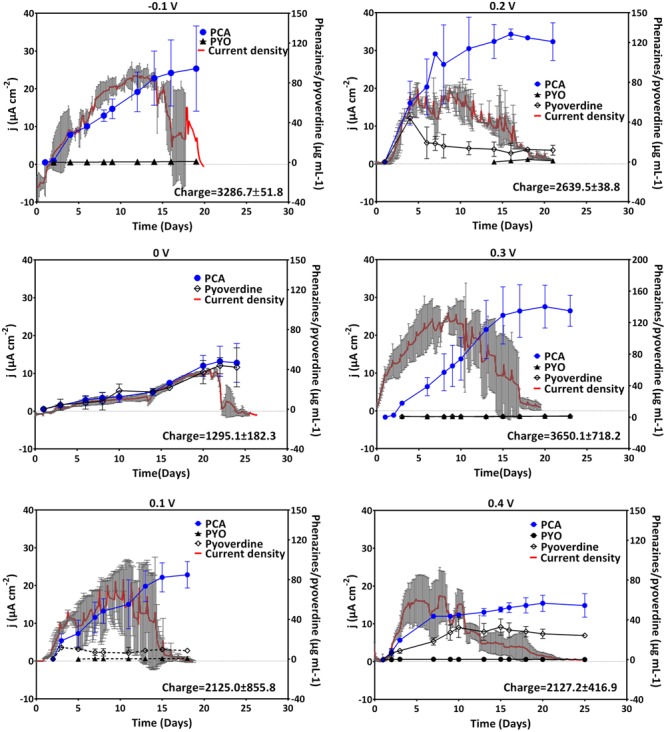
**Current generation and phenazine/pyoverdine production at anodic potentials.** Data for -0.1, 0, 0.1, and 0.2 V was obtained from two biological replicates whereas that of 0.3 and 0.4 was from three replicates; the mean current density and standard deviations are shown. Pyoverdines were only detected at 0, 0.1, and 0.4 V. Notice the different phenazines and pyoverdine axis scale for 0.3 V.

Anodic (oxidative) currents were observed at all potentials more positive than the formal potential of PCA (-0.1 to +0.4 V). A rapid positive increase in current reaching its maximum after 12 days was observed for cultures grown with an applied potential of -0.1 V as compared to -0.24 V (**Figure 2**). The elevated current production at -0.1 V coincided with very high amounts of PCA compared to the cathodic potentials described above (up to 94 μg mL^-1^ for -0.1 V). Averagely, except for 0 V, the maximum current densities generated at anodic potentials were fairly similar. At 0.4 V, maximum current density was attained earlier in the experiment compared to all other oxidative potentials, but then the current declined faster (after 10 days). An integration of the recorded current over time delivers the collected electric charge, whereby the most charge with about 3500 C was collected at -0.1 and 0.3 V. Except for 0 V, fairly similar charges (2200–2600 C) were collected at the other anodic potentials (**Figure 2**).

The observed current production reflects the production of phenazine redox mediators at the anodic potentials considered (**Figure 2**, right axes). Except for 0 V, elevated amounts of PCA were recorded at the anodic potentials; with cultures grown at -0.1, 0.2, and 0.3 V recording over 100 μg mL^-1^. In these cultures, averagely higher current densities were generated over a longer cultivation time. This was in line with the previous findings, where high concentrations of PCA were found to mediate elevated current production (Bosire et al., 2016). Consistent patterns of pyoverdine production could not be drawn.

### Cyclic Voltammetric Analysis

Cyclic voltammetry allows the analysis of the redox activity in the BES system. This may provide insight into the redox species employed in electron transfer by the biocatalysts. To decipher the use of redox species and the overall redox activity at different redox potentials, CV measurements were run by regularly (every 23 h) interrupting the potentiostatic current measurements. Redox peak systems obtained at the beginning of the experiment (blank), during rising activity and at the peak activity (in terms of electric current generation) of the culture were compared (Supplementary Figure S1). To obtain a meaningful interpretation of the redox species responsible for the peak systems, the cyclic voltammetric data was further compared to the phenazine quantification data of the time points of CV scans (**Table 1**).

**Table 1:** Formal potentials (E^° ′^) of the two peak systems observed at different applied electrode potentials, phenazine concentration, and pH.

	E^°′^		Phenazines (μg mL^-1^)^a^		
Potential	PS 1	PS 2	PCA	PYO	pH
–0.4	–0.197	0.064	32.5	0.1	6.30
–0.3	–0.164	0.042	75.2	0	6.42
–0.24	–0.195	0.049	5.05	0	6.39
–0.1	–0.24	0.05	89.97	0.71	6.35
0	–0.21	–^b^	48.0	0	6.24
0.1	–0.22	–^b^	82.24	0.5	6.33
0.2	–0.20	–^b^	12.61	3.2	6.20
0.3	–0.217	–^b^	129.22	0.82	6.47
0.4	–0.23	–^b^	43.63	0.42	6.42

*^a^Phenazine concentrations correspond to the day of CV record at peak current activity. ^b^Not observed.*

**FIGURE 3:**
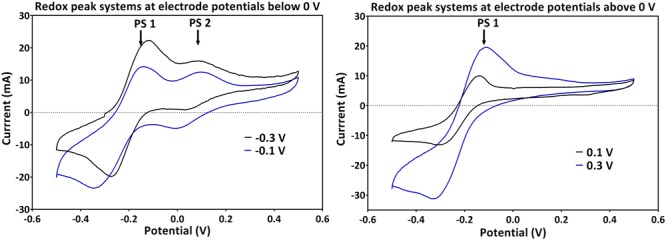
**Redox peak systems 1 and 2 (PS1 and PS2) observed for (left) applied electrode potentials below 0 V and (right) potentials above 0 V.** Two representative voltammograms obtained during the peak electric current generation are shown (for -0.1 and -0.3 V for below; and 0.1 and 0.3 V for above 0 V, respectively).

Generally across all applied potentials, two distinct redox peak systems were observed during the peak current production activity, which differed in the current intensity and the peak separation from each other (**Figure 3**). The mid peak potentials of the redox peak systems slightly shifted from one potential to the other (**Table 1**). Peak system 1 (PS1) was identified for all applied electrode potentials, while peak system 2 (PS2) was only detected for applied potentials lower than 0 V (**Figure 3** and **Table 1**).

The pH of all experiments was fairly similar (**Table 1**) and thus, cannot simply explain the potential shifts, which are more prominent at low applied electrode potentials. The mean formal potential of PS1 was the same as the formal potential of PCA standards [-0.24 V; (Bosire et al., 2016)]. Indeed, PCA was detected via HPLC as the dominating phenazine species in all experiments. The activity of PS1 can therefore be associated with the activity of PCA (**Figure 3** and **Table 1**). Changes in the chemical environment due to the different redox conditions in the experiments or due to adsorption processes to the electrode might have influenced the formal potential of PCA in the individual experiments.

**FIGURE 4:**
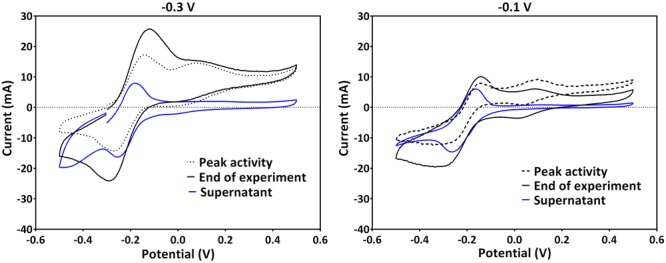
**A comparison of CVs obtained in the culture broth (with cells) and supernatants of the same cultures using clean electrodes for experiments poised at -0.3 V (left) and -0.1 V (right).** Representative voltammograms at the peak current activity, the end of the experiments and that of the supernatant are shown. Experiments at -0.3 V and -0.1 V were repeated to collect supernatant. Therefore, the CV traces at these potentials in this figure are not from the same experiments as in **Figure 3**.

Since PS2 had a positive formal potential (E°_1/2_ ∼ 0.052 V), which is not close to the E° of any *P. aeruginosa* phenazines, it is highly probable that this is not a peak system for a phenazine redox species (**Figure 3**). The performed HPLC analysis for phenazines and other hydrophobic compounds did not highlight any new, unidentified components. In an attempt to better characterize the compound behind PS2, a CV analysis was performed in the culture supernatants of the -0.3 and -0.1 V grown cultures using clean carbon electrodes. PS2 was not found in the supernatant CVs; hence, PS2 is a redox species that is likely adsorbed to the electrodes or located on the cells that form a biofilm around the electrode (**Figure 4**).

### Growth and Carbon Source Uptake

**FIGURE 5:**
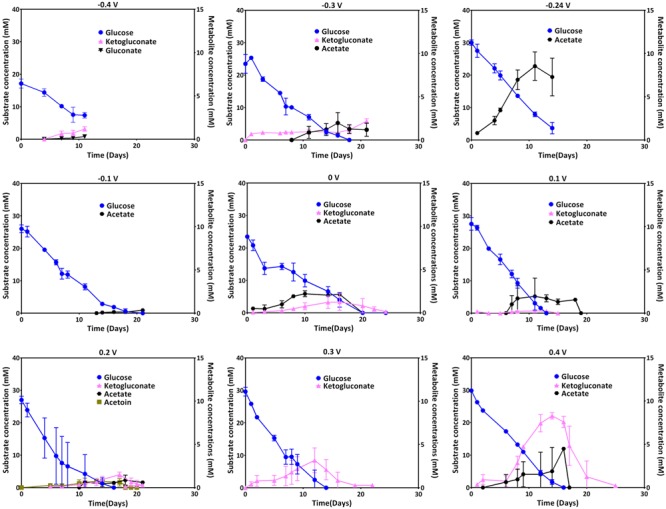
**Glucose uptake at different electrode potentials (left axis) and metabolite production (right axis; ketogluconate, gluconate, acetoin, and acetate) over time**.

Glucose uptake and the metabolites ketogluconate, gluconate, acetate, and acetoin were quantified during the culture period. The uptake of the carbon source during growth at most of the applied electrode potentials was complete by the end of each experiment (see individual experiments), except for -0.4 V where glucose was not depleted. At applied potentials below 0.1 V (especially at 0 V), the depletion of glucose took longer compared to the other applied potentials. Glucose uptake rates, were significantly higher for more positive electrode potentials (0.1 and -0.3 V), with the exception of 0.4 V where glucose consumption was slower again (**Table 2**). Instead, at this most positive electrode potential (0.4 V), elevated amounts of ketogluconate accumulated (∼8 mM). For all other potentials, ketogluconate levels – as an indicator for bottlenecks in the glucose consumption – stayed very low (<2 mM). Increased amounts of acetate (∼8 mM) were produced at -0.24 V compared to all other potentials applied (**Figure 5**).

**Table 2:** Influence of applied potential on the physiology of *Pseudomonas aeruginosa.*

	Potential (V)								
Parameter	–0.4	–0.3	–0.24	–0.1	0	0.1	0.2	0.3	0.4
j_max_ (μA cm^-2^)	–16.30 ± 1.09	–24.95 ± 6.33	–2.58 ± 0.24	22.6 ± 1.99	10.58 ± 0.51	17.8 ± 4.20	17.5 ± 1.57	23.94 ± 0.51	17.31 ± 4.83
Time of j_max_ (day)	11	9	8	12	21	10	9	8	5
Charge (Coulombs)	–1513.7 ± 30.7	–3409.7 ± 126.5	–823.4 ± 62.2	3286.6 ± 51.8	1295.1 ± 182.3	2125.0 ± 855.8	2639.5 ± 38.9	3650.1 ± 718.2	2127.2 ± 416.9
Coulombic efficiency (%)^a^	–	–	–	10.98 ± 0.27	4.30 ± 0.68	7.10 ± 2.86	7.38 ± 5.22	12.11 ± 3.36	7.08 ± 1.37
Biomass (g L^-1^ CDW)^b^	0.50 ± 0.13	0.81 ± 0.083	0.74 ± 0.08	0.55 ± 0.03	0.56 ± 0.05	0.57 ± 0.01	0.82 ± 0.31	0.72 ± 0.14	0.57 ± 0.02
Biofilm at electrode^c^	+	+++	+++	+++	+	++	+	+	+
PCA_max_ (μg mL^-1^)	38.3 ± 5.35	75.2 ± 7.42	7 ± 0.12	94.2 ± 30.20	48.0 ± 12.40	84.8 ± 10.90	128.4 ± 4.00	140.3 ± 22.20	56.7 ± 5.70
Pyoverdine (μg mL^-1^)	11.2	12.7 ± 2.74	0	0	43.8 ± 6.57	11.6 ± 1.60	44.1 ± 1.5	0	32.3 ± 5.77
Carbon source uptake (mM day^-1^)	0.98 ± 0.09	1.07 ± 0.11	1.91 ± 0.06	1.73 ± 0.05	0.96 ± 0.06	2.17 ± 0.06	2.302 ± 0.14	2.10 ± 0.1	1.88 ± 0.04

**FIGURE 6:**
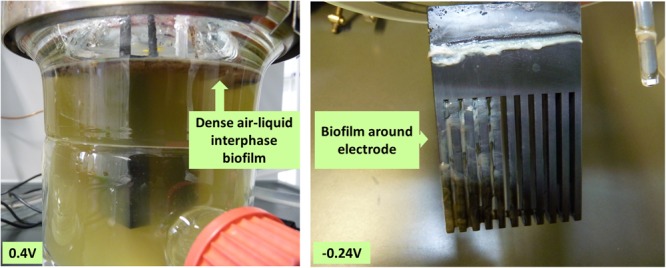
**Biofilm formation at the air-liquid interface and around the electrodes**. Representative pictures of biofilm formation: left 0.4 V, where a dense biofilm forms at the air-liquid-interphase and right -0.24 V, where a biofilm additionally forms around the electrode.

*^a^Percentage of the coulombs supplied in the substrate that were liberated as current. For cathodic currents coulombic efficiencies were not calculated (-). ^b^Biomass was determined from three replicate reactors, except -0.3, -0.1, 0, 0.1, and 0.2 V… -two replicates. ^c^Biofilm intensity was roughly visually determined around the electrode, with the following representations: + weak biofilm formation (barely visible), ++ medium biofilm formation (clearly visible), and +++ strong biofilm formation (thick biofilm such as in **Figure 6**-right).*

Growth during cultivation at different applied potentials was determined as total cell dry weight of the biofilm and planktonic cells at the end of the experiment (**Table 2**). Regular measurements of optical density as a measure for growth of *P. aeruginosa* is not reliable because of the strong biofilm formation tendency of this organism (especially at the air-liquid interface and around the electrodes; **Figure 6**). However, trends of total biomass generation at the different applied potentials were not clear. For instance, the most biomass was generated at 0.2 and -0.3 V. The unclear patterns in biomass generation would be partly due to the measurement of the biomass at the end of the culture experiment (time point at which current generation stopped), when most of the cells have attained the death phase. Also, the age of cultures from which the biomass was measured varied between the experiments (10–30 days); hence, the cultures measured were at different physiological states. Oxygen availability might have also influenced biomass generation since only passive aeration through open vent filters was applied, which might lead to stronger oxygen limitation in fast growing cultures.

Biofilm formation tendency was visually determined for all the cultures. At all potentials considered, a biofilm was formed at the air-liquid interphase at a considerably similar intensity. Cultures grown at negative applied potentials formed additionally a biofilm around the electrode at the liquid interphase. High biofilm intensity around the electrode was observed at -0.3, -0.24, and -0.1 V (**Figure 6** and **Table 2**).

To summarize our study, the overall influence of the applied potential on the current production, the redox species, and the growth was compiled in **Table 2**.

## Discussion

### Overall Influence of the Applied Electrode Potential on *P. aeruginosa* Physiology

The applied potential in a BES exerts an influence on the redox physiology of biocatalysts present (Busalmen et al., 2008; Wagner et al., 2010; Lian et al., 2016). *P. aeruginosa* might respond by fine tuning its electron transfer mechanism, e.g., by adjusting its spectrum or concentration of redox-active compounds. Furthermore, this might impact the central carbon metabolism and hence alter the overall respiration (Price-Whelan et al., 2007).

As stated above, there were no major differences observed in the biomass generation in cultures grown at the different potentials, except for the tendency to form a biofilm on the electrode at negative potentials (although a representative biomass determination was very difficult – see above). Theoretically, it would be possible that a beneficial use of higher electrode potentials of the extracellular electron acceptor, for instance 0.4 V for this study, would mediate higher biomass yields according to Gibbs energy (Thauer et al., 1977). However, this is only possible if different electron transfer mechanisms are available that can be employed at the different potentials for discharging electrons at these applied potentials. *P. aeruginosa*, however, uses mainly phenazines to reduce different external electron acceptors. Poising the electrode potential right at the formal potential of the most abundant phenazine (PCA) resulted in reduced production of this phenazine and the lack of a positive current, which is expected because current flow through this redox system is only possible if electron donor and acceptor to this system have a more negative and more positive potential, respectively, compared to its formal potential. High current densities across the potential range clearly coincided with high PCA concentrations, indicating that the dominating redox specie does not change with the applied electrode potential. For the organism, a productive usage of the PCA redox system is only possible at more positive potentials, which is also indicated by increased carbon source uptake rates at positive potentials as compared to negative potentials. Further, the reoxidation rate of the phenazines at the electrode should be higher (e.g., through enhanced electro-migration to the electrode), the more positive the electrode potential is compared to the formal potential of the redox species. In this sense, a potential of 0.3 V seems most appropriate for efficient PCA recycling. This is also reflected by the maximum observed coulombic efficiency, i.e., the fraction of chemical energy input that could be harvested at the anode, which was ∼12% at an applied potential of 0.3 V (**Table 2**). It appears though that increasing the potential above 0.3 V may not be beneficial for the organism, since the current production, the glucose uptake and the coulombic efficiency were all reduced at 0.4 V (**Figures 2, 5** and **Table 2**).

### PCA Can Also Be Involved in Cathodic Electron Transfer

At lower electrode potentials, the electron transfer to the electrode expectedly was impaired. Considering PCA (the most abundant phenazine) as the main electron transfer route to the electrode, it was expected that at and below -0.24 V no positive (anodic) current will be generated. Indeed at -0.24 very low amounts of PCA were detected and the cultures were barely electroactive. Instead, a cathodic reduction current was recorded at -0.24, -0.3, and -0.4 V. In this case, the electrode might have reduced the PCA redox mediator, which donated these electrons to suitable electron acceptors. Whether this reduction current was biologically utilized, is an important question to be answered. There is an indication that the current increased over time and reduced during the death phase, which might coincide with the growth of the culture and phenazine (PCA) production. Besides the microbial cells, also oxygen or minerals in the medium likely served as electron acceptor for these electrons in our study. It is widely accepted that *Pseudomonas* phenazines mediate electron transfer to distant oxygen in oxygen limited environments (Price-Whelan et al., 2006; Pierson and Pierson, 2010; Wang et al., 2010). Overall, below 0.1 V the oxidation of glucose is somewhat impaired; depicted in the reduced uptake rates, which might point to a lack of access to a required electron acceptor under these conditions, where the phenazine redox mediators are not available to discharge metabolic electrons to the electrode. This viewpoint is generally in agreement with our data, except for the experiment performed at -0.1 V, where high levels of PCA and the second highest levels of anodic currents were found. Identifying soluble redox species that can also mediate cathodic electron uptake is also an important question for current consuming BES applications such as microbial electrosynthesis or electrofermentation. For these systems, the mode of electron transfer from the cathode to a biocatalyst is still widely uncharacterized (Nevin et al., 2010, 2011; Moscoviz et al., 2016). This work indicates that PCA indeed can mediate cathodic electron flow, however, further work will have to show to what extend the mediated electrons flow to oxygen, minerals or into microbial processes.

### Unknown Redox Specie Involved in Cathodic Electron Transfer

Another important growth aspect in BES is the biofilm formation around the electrode. For BES microorganisms that depend on direct electron transfer, it is paramount that they are attached to the electrode. Even though *P. aeruginosa* is not known to depend on direct electron transfer, observations in this study suggest that negative potentials allow the formation of a biofilm around the electrode (to a higher degree at -0.24 V, see **Figure 6** and **Table 2**). Interestingly, at these potentials, an additional redox peak system (PS2) was observed with a more positive formal potential than that of PCA (0.052 V). Since the peak system was absent in the CVs of the supernatant, it is likely that PS2 is a redox species that is located at the electrode or the biofilm that formed around the electrode (**Figure 6**). Since this observation was only made for applied electrode potentials more negative than the formal potential of PS2, this may imply that *P. aeruginosa* employs additional redox species in maybe consuming electrons from the electrode. The electrons taken up from the electrode might be used to reduce other available electron acceptors for instance oxygen and minerals in the medium or feed into the microbial metabolism. It will be very interesting to further elucidate, which redox species is responsible for this additional redox peak system in future work and if this process represents a productive interaction of the microbial biocatalyst with the cathode. Overall, the results show that the applied potential influenced the electron transfer physiology, which might also be linked to carbon metabolism physiology. This implies that, for every BES investigations, it is important to determine the appropriate potential, which will be beneficial in steering the physiology of the biocatalyst for efficient current generation. From the data presented here, it can be concluded that 0.3 V was the most appropriate potential under the conditions of the BES used. 0.3 V allows a rapid generation of maximum current density, more charge and overall higher coulombic efficiency is attained. An applied anode potential of 0.3 V (∼0.5 V vs. SHE) is representative of the cell potential the bacteria would also encounter in a well-functioning MFC with an oxygen reduction cathode, where cell potentials are expected to reach 0.4–0.5 V. Thus, in such well-functioning MFCs, *P. aeruginosa* can find optimum electrochemical conditions to participate in electron transfer.

## Conclusion

*Pseudomonas aeruginosa* has demonstrated potential as a redox mediator producer for application in current generation in BES. To harness the full potential of the production of these mediators and their usage in electron shuttling, it is important to fully understand the influence of the applied electrode potential. This study reveals a profound influence of the applied potential on the levels and rate of current production for *P. aeruginosa* PA14 at different electrode potentials. Higher potentials (up to 0.3 V) increase the rate of anodic peak current generation, while potentials more negative than the formal potential of the dominating redox specie result in cathodic electron uptake. Thereby, the redox mediator PCA was identified as the dominating redox specie at all tested potentials. Further, *P. aeruginosa* activates an additional redox species at lower potentials, of which activity and specific role is yet to be identified.

## Author Contributions

EB designed the work, conducted the experiments, analyzed the data, and prepared the draft of the manuscript. MR conceived of the study, designed the work, analysed the data, and edited the manuscript.

## Conflict of Interest Statement

The authors declare that the research was conducted in the absence of any commercial or financial relationships that could be construed as a potential conflict of interest.
