# Supplementary material for: Electrochemical Potential Influences Phenazine Production, Electron Transfer and Consequently Electric Current Generation by Pseudomonas aeruginosa
Source: Front Microbiol. 2017 May 18;8:892. doi: 10.3389/fmicb.2017.00892 (PMC5435749; doi:10.3389/fmicb.2017.00892)
Supplement: Supplementary file 1 [file Data_Sheet_1.DOCX]

Supplementary Material

Electrochemical Potential Influences Phenazine Production, Electron Transfer and Consequently Electric Current Generation by *Pseudomonas aeruginosa*

Erick M. Bosire^1^, Miriam A. Rosenbaum^1*^

^1^Institute of Applied Microbiology – iAMB, Aachen Biology and Biotechnology – ABBt, RWTH Aachen University, Aachen, Germany

*** Correspondence:**Miriam A. Rosenbaum
miriam.rosenbaum@rwth-aachen.de

## Supplementary Figures:

**
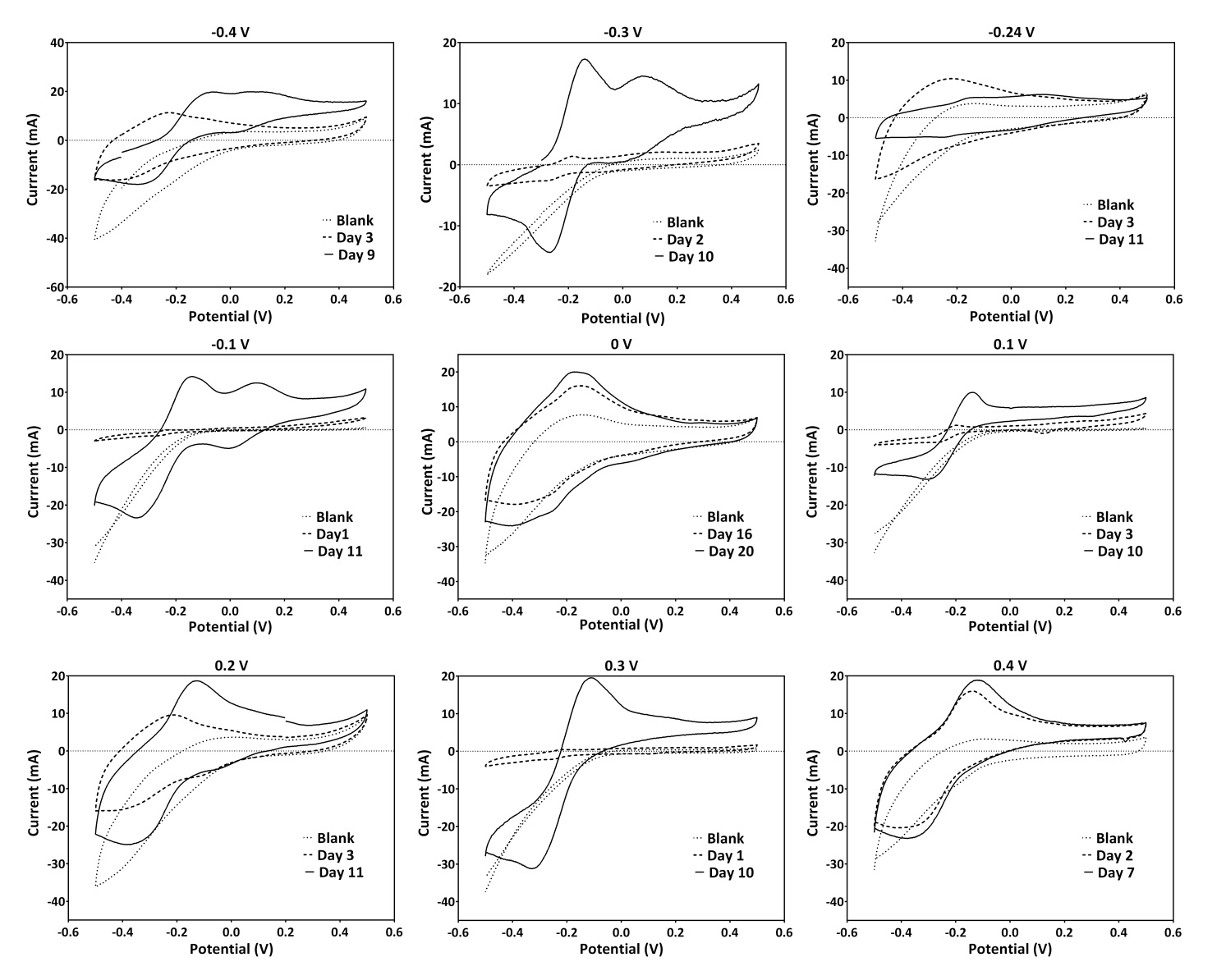
**

**Supplementary Figure S1: Cyclic voltammograms for cultures grow at (top) -0.4 V, -0.3 V, -0.24, (middle) -0.1 V, 0 V, 0.1 V and (bottom) 0.2 V, 0.3 V, 0.4. They include a blank CV taken before inoculation, one taken at early stages of the culture growth and one at the peak current generation activity.**
